# Long intergenic noncoding RNA00265 promotes proliferation of gastric cancer via the microRNA-144-3p/Chromobox 4 axis

**DOI:** 10.1080/21655979.2021.1876320

**Published:** 2021-03-29

**Authors:** Zengxi Yang, Xi OuYang, Liang Zheng, Lizhen Dai, Wenjuan Luo

**Affiliations:** a Department of General Surgery, Lanzhou University Second Hospital, Lanzhou, China; b Department of Gastrointestinal Surgery, The Second Affiliated Hospital of Nanchang University, Nanchang, China; c Department of Obstetrics, The Third Affiliated Hospital of Sun Yat-sen University, Guangzhou, China; d Department of Ophthalmology, Lanzhou University Second Hospital, Lanzhou, China

**Keywords:** LINC00265, gastric cancer, competing endogenous RNA, microRNA-144-3p

## Abstract

The expression and biological function of long intergenic noncoding RNA00265 (LINC00265) in gastric cancer (GC) have not yet been explored. This study aimed to detect LINC00265 expression in GC tissues and cell lines, investigate its roles in the proliferation of GC cells in vitro, and elucidate the regulatory mechanisms of LINC00265 action. It was found that LINC00265 expression was significantly upregulated in GC tissue samples and cell lines compared with their normal counterparts. Additionally, LINC00265 knockdown could inhibit GC cell proliferation in vitro. Further investigation revealed that LINC00265 acted as a competing endogenous RNA for microRNA-144-3p (miR-144-3p) and inhibition of miR-144-3p markedly counteracted LINC00265 knockdown-meditated suppression on GC cell proliferation. Additionally, Chromobox 4 (CBX4) was upregulated in GC and silencing CBX4 could reduce GC cell proliferation. Then, CBX4 mRNA was demonstrated to be a direct target of miR-144-3p in GC cells and LINC00265/miR-144-3p axis could regulate CBX4 expression. Taken together, LINC00265 may promote GC cell proliferation via the miR-144-3p/CBX4 axis.

## Graphical Abstract

**Figure:**
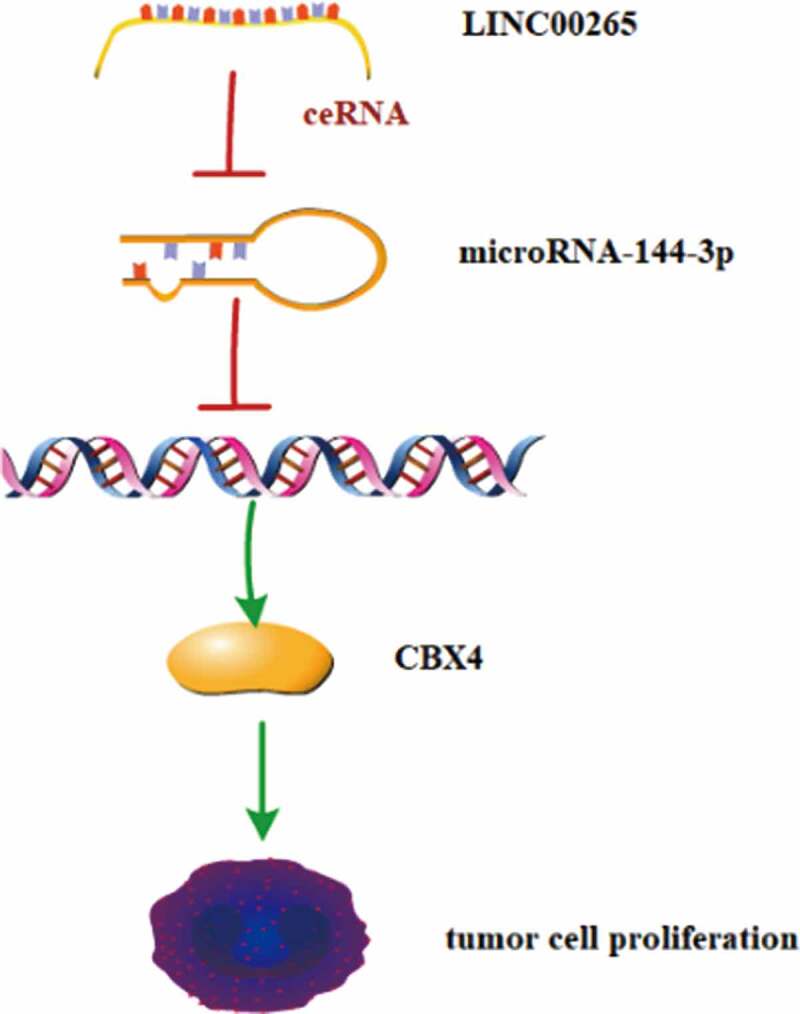

## Introduction

Gastric cancer is one of the most common malignant cancer in the world and ranks the third most common causes of cancer mortality worldwide^[1]^. The incidence rate has been dramatically increasing in Eastern Asia during the past decades [2,3]. Because GC exhibits no specific symptoms at early stage and sensitive biomarkers for GC are not available currently, GC patients are always diagnosed at advanced stage, especially in China [4,5]. Even worse, current treatment options for this devastating disease are extremely limited [6]. As result, GC patients at advanced stage have rather poor long-term survival outcome. Therefore, it is urgent to further reveal the molecular mechanisms underlying the occurrence and development of gastric cancer [7], in order to develop novel therapeutic methods.

There are many risk factors contributing to the incidence and development of GC, such as Helicobacter pylori (H.pylori) infection, dietary habits, tobacco use, and obesity [8]. Helicobacter pylori is a well-known risk factor for GC and played an important pro-tumor role in GC. The mechanisms underlying the pro-tumor role of in H.pylori are rather complex. For example, gastritis caused by H.pylori infection could change the immune environment to induce carcinogenesis, and H.pylori could also directly act on epithelial cells to induce gene mutation, which aberrantly activates tumor-associated signaling pathways, ultimately resulting GC progression [9,10]. Interestingly, growing evidence suggests that H.pylori infection could contribute to GC tumorigenesis by regulating lncRNAs. Han et al. suggested that H.pylori infection could upregulate lncRNA SNHG17 in GC cells. Furthermore, they uncovered that lncRNA SNHG17/NONO and SNHG17/miR-3909/RING1/Rad51 pathways could accelerate GC tumorigenesis through disturbing the DNA repair system [11]. In addition, a recent study indicated that H.pylori could promote GC cell growth and migration by upregulating THAP9-AS1 [12].

LncRNAs are a class of non-coding RNAs with longer than 200 nucleotides and their dysregulations can promote cancer cell proliferation, metastatic cascades, and chemoradiotherapy resistance via post-transcription regulatory mechanisms, such as acting as miRNA sponge [13–15]. Dysregulation of lncRNA occurs frequently in GC and closely correlates with disease progression [16]. For example, lncRNA KCNQ1OT1 was found to be downregulated in GC and it could function as a competing endogenous RNA for microRNA-9 (miR-9) to regulate LMX1A expression, consequently repressing GC cell proliferation [17]. Besides, a recent study indicated that lncRNA PVT1 was highly expressed in GC tissues and can promote tumor progression by interacting with FoxM1, and PVT1 [18]. Furthermore, Xiao et al. reported that lncRNA TRPM2-AS exerted a pro-tumor effect by sponging miR-612 in GC cells [19]. In addition, there are many other dysregulated lncRNAs involved in GC progression, such as LINC00152, PANDA, MAGI2-AS3, PWRN1 and TUBA4B [20]. With the development and application of high throughput sequence technology, more and more novel lncRNAs dysregulated in cancers are identified. Recently, LINC00265 was shown to be upregulated in colorectal cancer (CRC) and it could enhance cell viability and glycolysis through negatively regulating miR-216b-5p [21]. Additionally, it has been demonstrated that LINC00265 was highly expressed in bone marrow and serum of acute myeloid leukemia (AML) patients, and it could promote AML cell proliferation and migration by activating PI3K/AKT signaling pathway [22]. However, no information regarding the role of LINC00265 in GC is not available currently.

MicroRNAs (miRNAs) are noncoding RNAs 20–24 nt in length, which have the ability to modulate the stability and translational efficiency of targeted mRNAs [23]. Solid evidence has demonstrated that miRNAs participate in various biological processes, such as cell proliferation, differentiation, apoptosis and metabolism [24]. Notably, numerous studies indicated that a multiple of miRNAs are dysregulated in many cancer types, including GC [25]. Particularly, Faheim M. et al revealed that miR-144 was downregulated in GC cells, and miR-144 could inhibit GC cell proliferation and invasion by directly targeting activating enhancer-binding protein 4 (AP4) [26]. Yao. et al. showed that miR-144 could negatively regulate the expression of cyclooxygenase-2 (COX-2) to repress GC cell viability [27]. However, the mechanisms responsible for low miR-144 expression and pro-tumorigenic mechanisms in GC are not completely deciphered currently.

Chromobox family, a subgroup of proteins in the PcG family, has eight members including CBX1-8. These proteins display distinct biological functions in different tissues. Chromobox 4 (CBX4), also known as polycomb 2 (Pc2), is a special chromobox protein, since it is not only a transcriptional repressor but also a SUMO E3 ligase [28]. Increasing evidence suggested that CBX4 might be an oncogene and could be exploited as a therapeutic target in several cancers, including lung cancer, cervical cancer, breast cancer and osteosarcoma [29–31]. For example, CBX4 could promote the proliferation and metastasis of lung cancer cells in vitro through enhancing BMI-1-mediated expression and activation of P53, CDK2, Cyclin E, MMP2, MMP9 and CXCR4 [32]. However, CBX4 was found to play a tumor suppressor in colorectal carcinoma via recruitment of HDAC3 to the runx2 Promoter [33]. Therefore, whether CBX4 functions as an oncogene or tumor suppressor may depend on the genetic context of cancer. To date, the role of CBX4 in gastric cancer has never been studied.

In this study, we initially evaluated the expression status of LINC00265 in GC tissues and cell lines, and explored its effect on GC cell proliferation and the corresponding mechanisms, so as to find the potential therapeutic targets and prognostic biomarkers. Our data suggest that LINC00265 is overexpressed in GC and exerts oncogenic activities via the miR-144-5p/CBX4 signaling pathway.

## Material and methods

### Ethics statement

Investigation was conducted in compliance with the principles of the Declaration of Helsinki and according to national and international guidelines. Moreover, this study protocol was approved by the Ethics Committee of The Second Affiliated Hospital of Nanchang University. Informed consent has been obtained from all patients.

### Cell lines

GC cell lines (KATO-III, SGC-7901, BGC-823, HGC-27, AGS, NCI-N87, SNU-1 and SNU-16) and normal gastric epithelial cells (GES cells) were bought from the Shanghai Institute of Biochemistry and Cell Biology (Shanghai, China). All the cells were cultured at 37°C in a humidified atmosphere containing 5% of CO2 in Dulbecco’s Modified Eagle’s Medium (DMEM) supplemented with 10% of fetal bovine serum (FBS),100 U/ml penicillin, and 100 µg/ml streptomycin (all from Gibco, Invitrogen Life Technologies, Carlsbad, CA, USA).

#### Oligonucleotides, construction of plasmids, and cell transfection

The siRNA specifically decreasing LINC00265 expression (called si-LINC00265) and its negative control siRNA (si-NC) were designed and commercially synthesized by RiboBio Co., Ltd. (Guangzhou, China). AgomiR-144-3p, the corresponding agomir-NC, antagomiR-144-3p and antagomir-NC were acquired from the GenePharma Co., Ltd. (Shanghai, China). The full length CBX4 sequence lacking its 3′-UTR was amplified by the GenePharma Co., Ltd., too, and subcloned into the pcDNA3.1 vector to generate the pcCBX4 plasmid. The empty pcDNA3.1 vector served as the control. Cells were seeded in 6-well plates and transfected with the agomir (50 nM), antagomir (50 nM), siRNA (100 pmol) or plasmid (4 µg) using the Lipofectamine® 2000 reagent (Invitrogen; Thermo Fisher Scientific, Inc.). The transfected cells were processed for further in vitro experiments after incubation for different periods.

#### RNA isolation and reverse-transcription quantitative PCR

The TRIzol® Reagent (Invitrogen; Thermo Fisher Scientific, Inc.) was used to extract total RNA from tissue samples or cells. The miScript Reverse Transcription Kit was purchased from Qiagen GmbH (Hilden, Germany), and then used for the synthesis of cDNA from the total RNA. After reverse transcription, reverse-transcription quantitative PCR (RT-PCR) was performed using the miScript SYBR Green PCR Kit (Qiagen GmbH) to determine miR-144-3p expression. The expression level was determined in relation to U6 small nuclear RNA. To quantitate LINC00265 and CBX4 expression, the synthesis of cDNA was performed using the PrimeScript RT-Reagent Kit (Takara Bio, Kusatsu, Japan), followed by RT-PCR with the SYBR Premix Ex Taq™ Kit (Takara Bio). Expression levels of LINC00265 and CBX4 were normalized to GAPDH. All the data were analyzed by the 2^−ΔΔCq^ method.

### CCK-8 assay and colony formation assay

After 24 h transfection, cells were maintained in 96-well plates in 10% FBS-supplemented DMEM for 0, 24, 48, or 72 h. At every time point, 10 µl of the CCK-8 solution (Dojindo Laboratories, Kumamoto, Japan) was added into each well, and the cells were incubated further at 37°C for 2 h. Optical density was measured at 450 nm wavelength on a Sunrise™ microplate reader (Tecan Group, Ltd., Mannedorf, Switzerland). As for colony formation assay, GC cells were gathered and maintained in in 6-well plates after 24 h transfection. Then, the cells were cultured with complete medium at 37°C with 5% CO2 for half a month. On day 15, GC cells were fixed in 4% paraformaldehyde and stained using 0.5% crystal violet. At the last step, the colonies were counted under microscope.

### Nuclear/cytoplasmic fractionation

Briefly, cells were washed twice with cold PBS, and lysed in cell lysis buffer (20 mmol/L Tris-HCl pH 8, 0.5% NP40, 10 mmol/L NaCl, 3 mmol/L MgCl2 supplemented with protease inhibitors) for 30 min on ice with vigorous pipetting every 5 min. Nuclei were sedimented by centrifugation at 5000 × g for 5 min at 4°C and the supernatant (cytoplasmic fraction) was collected. The nuclear pellet was washed 3 times in cell lysis buffer to eliminate cytoplasmic traces and the nuclei were lysed in nuclear lysis buffer (50 mmol/L Tris-HCl, 1% SDS) on ice for 20 min followed by brief sonication to ensure nuclei rupture. Nuclear debri was collected by centrifugation at 12,500 × g for 10 min at 4°C and the supernatant (nuclear fraction) was gathered. Nuclear and cytoplasmic fractions were stored at −80°C prior to analysis by SDS-PAGE and western blotting.

### Bioinformatics prediction and luciferase reporter assay

A target prediction tool, starBase 3.0 (http://starbase.sysu.edu.cn/), was used to predict potential miRNAs targeted by LINC00265. The potential targeted mRNAs of miR-144-3p were predicted using two miRNA target prediction databases: starBase 3.0 and TargetScan (http://www.targetscan.org/). The CBX4 3′-UTRs containing either the wild-type (wt) binding sequence or the mutant (mut) binding sequence for miR-144-3p were synthesized by GenePharma Co., Ltd., and inserted into the pmirGLO luciferase reporter vector (Promega, Madison, WI, USA). The resultant plasmids are referred to as CBX4-wt and CBX4-mut, respectively. The LINC00265-wt and LINC00265-mut reporter plasmids were produced by similar experimental procedures. Either agomiR-144-3p or agomir-NC was co-transfected into cells with either a ‘wt’ or ‘mut’ reporter plasmid using the Lipofectamine® 2000 reagent. Luciferase activity was determined 48 h after transfection using a Dual-Luciferase Reporter Assay System (Promega, Madison, WI, USA). *Renilla* luciferase activity was normalized to that of firefly luciferase.

### Western blotting analysis

Total protein was isolated by means of the RIPA buffer (Beyotime Institute of Biotechnology, Shanghai, China). The Bicinchoninic Acid Protein Assay Kit (Beyotime Institute of Biotechnology, Shanghai, China) was used to determine the protein concentration. The protein samples were resolved by SDS-PAGE on a 10% gel and transferred onto polyvinylidene difluoride membranes, followed by 2 h blocking with 5% fat-free milk diluted in Tris-buffered saline containing 0.1% of Tween 20 (TBST). After incubation with a primary antibody against CBX4 (cat. 18,544-1-AP; dilution 1:500; proteintech) or against GAPDH (cat. 60,004-1-Ig; dilution 1:5000; proteintech), the membranes were washed thrice with TBST, probed with a goat anti-rabbit immunoglobulin G antibody conjugated with horseradish peroxidase (cat. No. ab205718; dilution 1:5,000; Abcam) (secondary antibody) and, then, treated with the Pierce™ ECL Western Blotting Substrate (Pierce; Thermo Fisher Scientific, Inc.) for visualization of the protein signals.

### Statistical analysis

Data were analyzed using SPSS version 13.0 software (SPSS; Chicago, USA). Data are expressed as the mean ± standard deviation (SD) of at least three independent experiments. The comparisons between two groups were performed by Student’s t test; one-way analysis of variance, followed by the Student-Newman-Keuls test, was applied to evaluate the differences among multiple groups. Data with P < 0.05 were considered statistically significant.

## Results

Growing evidence has demonstrated that many lncRNAs are dysregulated in GC and play important roles in promoting disease progression [33], suggesting these lncRNAs may be potential diagnostic biomarkers and therapeutic targets for GC. Previous studies reported that LINC00265 was highly expressed in CRC and AML, and performed oncogenic functions [21,22]. However, LINC00265 expression and its role in GC have never been explored yet. In this study, we thus detected LINC00265 expression in GC, clarified its effect on GC cell proliferation in vitro and clarified the underlying mechanisms, in order to determine whether LINC00265 could be exploited as the potential therapeutic target and prognostic biomarker for GC.

### LINC00265 is overexpressed in GC tissue samples and cell lines

**Figure 1.**
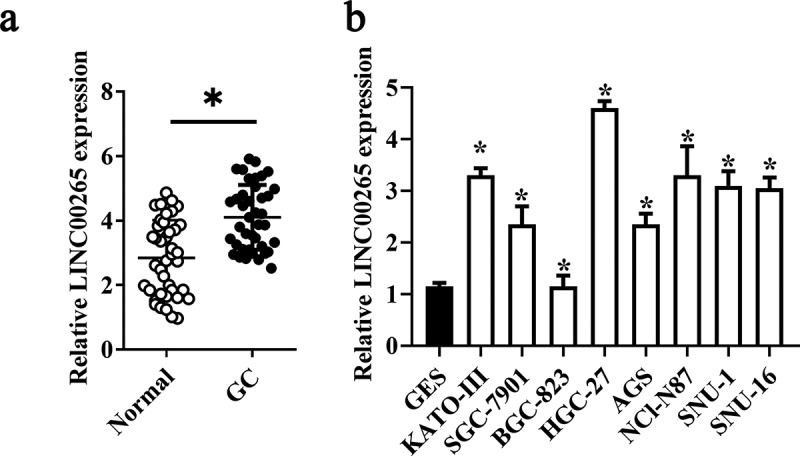
**LINC00265 is overexpressed in GC tissue samples and cell lines**. (a) The expression of LINC00265 was analyzed in 32 pairs of GC tissue samples and adjacent normal gastric tissues using RT-qPCR. *P < 0.05 vs. the normal gastric tissues. (b) RT-qPCR was performed to determine LINC00265 expression in eight GC cell lines (KATO-III, SGC-7901, BGC-823, HGC-27, AGS, NCI-N87, SNU-1, and SNU-16) and normal gastric cells (GES cells). *P < 0.05 vs. GES cells

To determine the role of LINC00265 in GC, we first examined its expression profile in 32 pairs of GC tissue samples and adjacent normal tissue samples using RT-PCR. LINC00265 was found to be overexpressed in the GC tissue samples relative to the adjacent normal gastric tissues (Figure 1a, P < 0.05). Moreover, it was also found that LINC00265 expression was significantly higher in a panel of GC cell lines (KATO-III, SGC-7901, BGC-823, HGC-27, AGS, NCI-N87, SNU-1, and SNU-16) compared with that in normal gastric cells (GES cells) (Figure 1b, P < 0.05). These results implied that LINC00265 is upregulated in GC.

### LINC00265 knockdown inhibits GC cell growth in vitro

**Figure 2.**
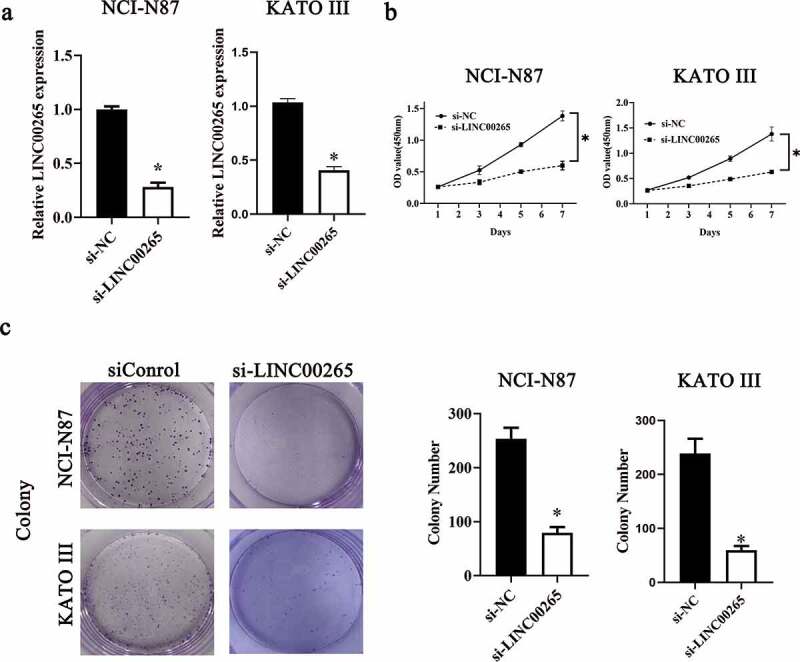
**LINC00265 knockdown inhibits GC cell growth in vitro**. (a) NCI-N87 and KATO III cells were transfected with either si-LINC00265 or si-NC. At 48 h post-transfection, the cells were collected and, then, subjected to RT-qPCR analysis for transfection efficiency evaluation. *P < 0.05 vs. the si-NC group. (b) The CCK-8 assay was conducted to assess cellular proliferation after 0, 3, 5, and 7 day of cultivation of si-LINC00265-transfected or si-NC-transfected NCI-N87 and KATO III cells. *P < 0.05 vs. group si-NC. (c) Colony formation assay was performed to determine the proliferation ability of NCI-N87 and KATO III cells after transfection with either si-LINC00265 or si-NC. *P < 0.05 vs. the si-NC group

Considering the aberrant upregulation of LINC00265 in GC, we next attempted to determine the functions of LINC00265 in GC progression. NCI-N87 and KATO III cells were chosen for subsequent experiments and were transfected with either a small interfering RNA (siRNA) against LINC00265 (si-LINC00265) or a negative control siRNA (si-NC). LINC00265 was successfully knocked down in NCI-N87 and KATO III cells after transfection of si-LINC00265 (Figure 2a, P < 0.05). The Cell Counting Kit-8 (CCK-8) assay was performed to evaluate the influence of si-LINC00265 on GC cell proliferation. The si-LINC00265 transfection obviously reduced the proliferative ability of NCI-N87 and KATO III cells compared with si-NC transfection (Figure 2b, P < 0.05). Then, colony formation assay was conducted to further confirm whether LINC00265 knockdown inhibit GC cell growth in vitro. As expected, the colony number of NCI-N87 and KATO III cells was significantly reduced after transfection with si-LINC00265 (Figure 2c, P < 0.05). In general, these findings suggested that LINC00265 knockdown could inhibit GC cell growth in vitro.

### LINC00265 promotes GC cell growth by inhibiting miR-144-3p as a competing endogenous RNA (ceRNA)

**Figure 3.**
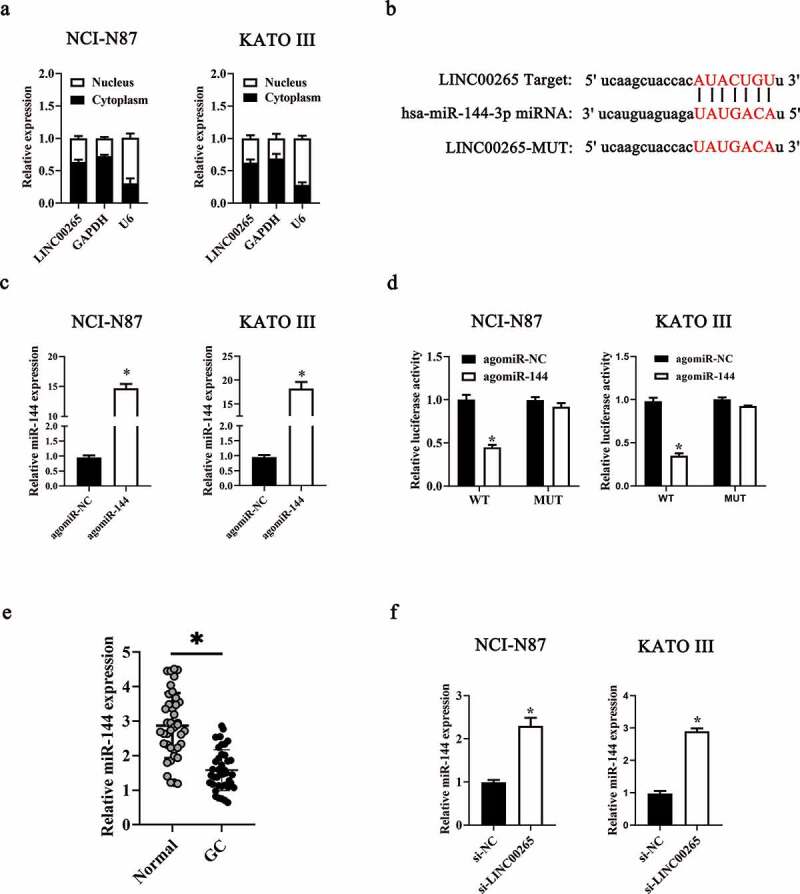
**LINC00265 functions as a ceRNA for miR-144 in GC cells**. (a) The distribution of LINC00265 within GC cells was determined by the nuclear/cytoplasmic fractionation assay. (b) The wild-type miR-144-binding sequences in LINC00265, as predicted by starBase 3.0. The mutations in the LINC00265 sequence that disrupt the interaction between LINC00265 and miR-144 are shown too. (c) NCI-N87 and KATO III cells that were transfected with either agomiR-144 or agomir-NC were harvested and analyzed for miR-144 expression by RT-qPCR. *P < 0.05 vs. the agomir-NC group. (d) Luciferase reporter assays were performed on NCI-N87 and KATO III cells that were transfected with either agomiR-144 or agomir-NC and either LINC00265-wt or LINC00265-mut. *P < 0.05 vs. group agomir-NC. (e) The expression profile of miR-144 in the 40 pairs of GC tissues and adjacent-normal-gastric tissue samples was analyzed by RT-qPCR. *P < 0.05 vs. the normal tissues. (f) Expression of miR-144 in NCI-N87 and KATO III cells transfected with either si-LINC00265 or si-NC was determined by RT-qPCR. *P < 0.05 vs. the si-NC group

**Figure 4.**
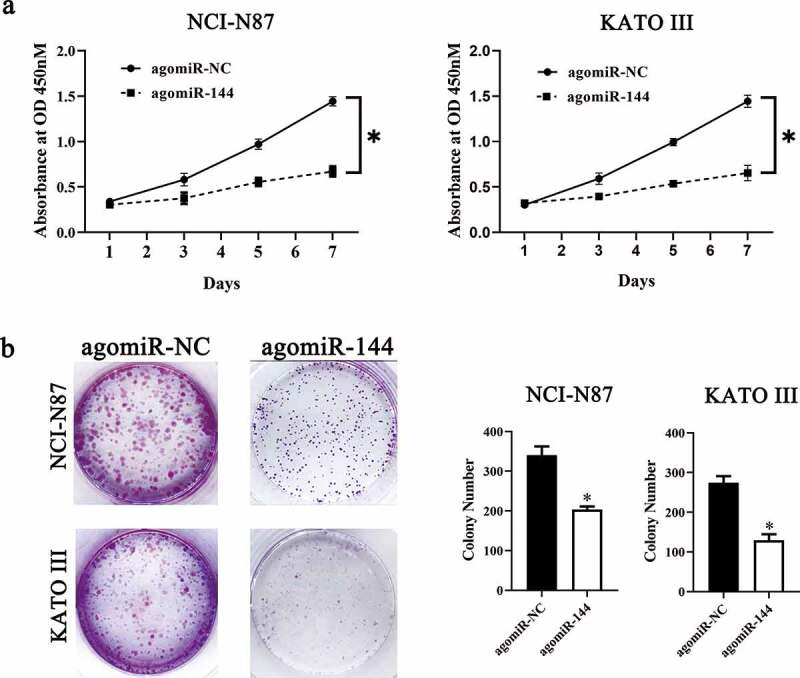
**MiR-144 exerts a suppressive effect on GC cell growth in vitro**. (a) The CCK-8 assay uncovered a change in proliferation of miR-144-overexpressing NCI-N87 and KATO III cells. *P < 0.05 vs. group agomir-NC. (b) NCI-N87 and KATO III cells were treated with either agomiR-144 or agomir-NC. After the transfection, Colony formation assays were carried out. *P < 0.05 vs. group agomir-NC

To investigate the molecular events involved in LINC00265-mediated GC proliferation, a nuclear/cytoplasmic fractionation assay was first conducted to determine the distribution of LINC00265 in GC cells. The data indicated that LINC00265 was mainly located in the cytoplasm of GC cells (Figure 3a), which suggested that this lncRNA may serve as a ceRNA for some miRNA(s) [34]. Then, starBase 3.0. was employed to predict the candidate miRNAs probably inactivated by LINC00265. The result showed that LINC00265 contained one conserved binding site for miR-144-3p (Figure 3b). Luciferase reporter was performed to further characterize the relation between LINC00265 and miR-144-3p in GC cells. MiR-144-3p agomir (agomiR-144-3p) transfection-mediated upregulation of miR-144-3p (Figure 3c, P < 0.05) noticeably decreased the luciferase activity of the LINC00265-WT plasmid (the plasmid expressing LINC00265 containing the wild-type binding site for miR-144-3p; P < 0.05) in both NCI-N87 and KATO III cells; however, the luciferase activity of LINC00265-MUT (the plasmid expressing LINC00265 containing a mutant binding site for miR-144-3p) was unaffected when miR-144-3p was overexpressed, as evidenced by the luciferase reporter assay (Figure 3d). These results suggested that miR-144-3p was a target of LINC00265 in GC cells. Next, we detected miR-144-3p expression in the 40 pairs of GC tissue samples and adjacent normal gastric tissues by RT-qPCR. MiR-144-3p was found to be significantly downregulated in the GC tissue samples (Figure 3e, P < 0.05). Furthermore, the expression of miR-144-3p was evaluated in LINC00265-deficient NCI-N87 and KATO III cells. The LINC00265 knockdown remarkably upregulated miR-144-3p in NCI-N87 and KATO III cells (figure 3f, P < 0.05). Strikingly, previous studies suggested that miR-144-3p functioned as a tumor suppressor in GC [35,36]. Consistently, in this study it was found that transfection with agomiR-144-3p appreciably repressed proliferation (Figure 4a&B, P < 0.05) of NCI-N87 and KATO III cells. In general, these results indicated that LINC00265 promoted GC cell proliferation by inhibiting miR-144-3p as a ceRNA.

### LINC00265 sponges miR-144-3p to upregulate CBX4 expression in GC cells

**Figure 5.**
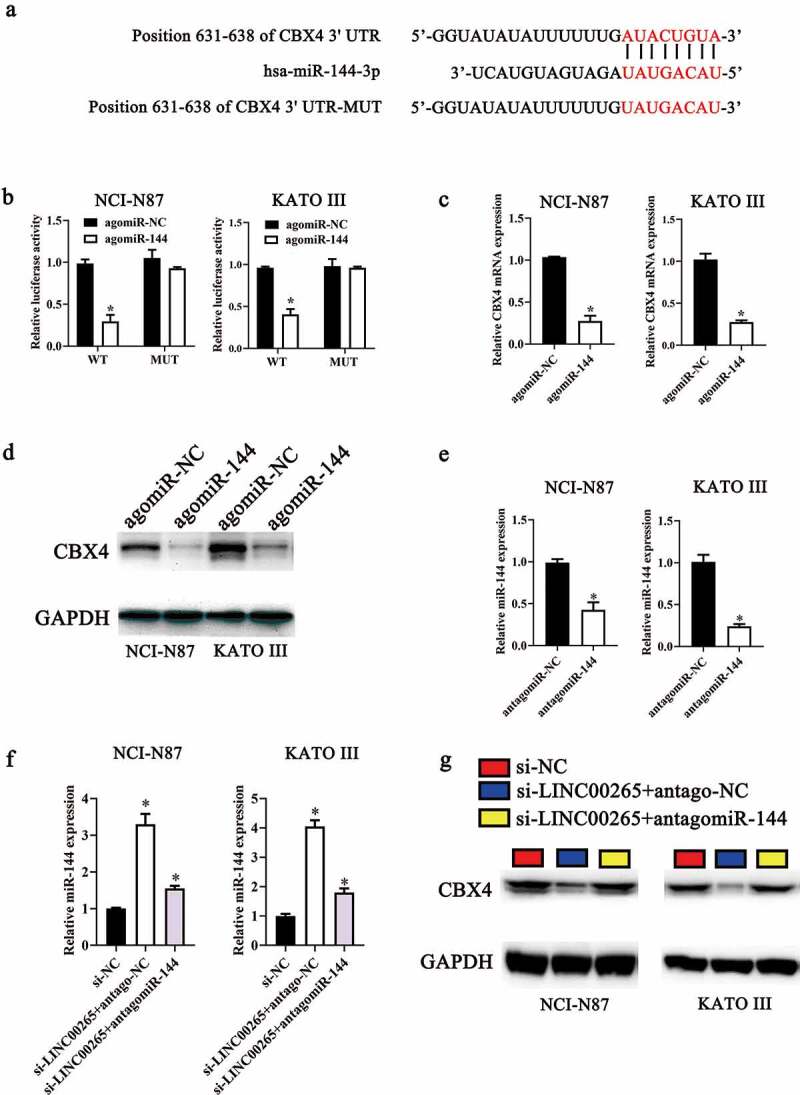
**LINC00265 sponges miR-144-3p to upregulate CBX4 expression in GC cells**. (a) MiR-144 and its wild-type binding site in the 3′-UTR of CBX4 mRNA. The mutations were introduced into the site complementary to the seed region of miR-144. (b) The luciferase reporter assay was performed to test whether the 3′-UTR of CBX4 mRNA could be directly targeted by miR-144 in GC cells. NCI-N87 and KATO III cells were cotransfected with either agomiR-144 or agomir-NC and either the CBX4-wt or CBX4-mut plasmid. After 48 h of cultivation, the transfected cells were assayed with the Dual-Luciferase Reporter Assay System to measure the luciferase activity. *P < 0.05 vs. the agomir-NC group. (c, d) Expression levels of CBX4 mRNA and protein in miR-144-overexpressing NCI-N87 and KATO III cells were respectively determined by RT-qPCR and western blotting. *P < 0.05 vs. the agomir-NC group. (e) Expression levels of miR-144 in NCI-N87 and KATO III cells transfected with antagomiR-144 or antagomir-NC. (f, g) Si-LINC00265 in combination with either antagomiR-144 or antagomir-NC was transfected into NCI-N87 and KATO III cells. After 48 h transfection, expression levels of the CBX4 protein and miR-144 were determined respectively by western blotting and RT-qPCR. *P < 0.05 vs. group si-NC. #P < 0.05 vs. group si-LINC00265+ antagomir-NC

We next continued to ask how LINC00265/miR-144-3p axis regulated GC cell growth in vitro. To this end, we first employed starBase 3.0 and TargetScan to predict the potential targets of miR-144-3p. CBX4 was predicted as a potential target gene of miR-144-3p (Figure 5a). To validate this prediction, a luciferase reporter assay was performed on NCI-N87 and KATO III cells after cotransfection with either agomiR-144-3p or agomir-NC and either plasmid CBX4-WT (a plasmid expressing luciferase mRNA containing the CBX4 3′-UTR harboring a wild-type binding site for miR-144-3p) or plasmid CBX4-MUT (a plasmid expressing luciferase mRNA containing the CBX4 3′-UTR harboring a mutated binding site for miR-144-3p). The ectopic expression of miR-144-3p significantly reduced the luciferase activity of CBX4-WT in NCI-N87 and KATO III cells (P < 0.05). By contrast, mutation of the binding site abrogated this phenomenon (Figure 5b). Next, the expression levels of CBX4 in miR-144-3p-overexpressing NCI-N87 and KATO III cells were determined to investigate whether miR-144-3p could target CBX4 in GC cells. As expected, the protein levels (Figure 5c, P < 0.05) and mRNA (Figure 5d, P < 0.05) of CBX4 in NCI-N87 and KATO III cells diminished in response to the agomiR-144-3p transfection. Collectively, these results identified CBX4 mRNA as a direct target of miR-144-3p in GC cells.

Then, to further determine whether LINC00265 sponged miR-144-3p to upregulate CBX4 expression in GC cells, we transfected LINC00265-deficient NCI-N87 and KATO III cells with miR-144-3p antagomir (antagomiR-144-3p). As shown in Figure 5e, transfection of antagomiR-144-3p efficiently increased miR-144-3p expression in NCI-N87 and KATO III cells. However, miR-144-3p expression levels were relatively downregulated after co-transfection with si-LINC00265 and antagomiR-144-3p (figure 5f, P < 0.05). Furthermore, CBX4 expression was downregulated in NCI-N87 and KATO III cells co-transfected with si-LINC00265 and antagomiR-NC, while CBX4 expression was markedly recovered by co-transfection with si-LINC00265 and antagomiR-144-3p (Figure 5g, P < 0.05). Taken together, these data revealed that LINC00265 sponged miR-144-3p to upregulate CBX4 expression in GC cells, suggesting LINC00265 knockdown may inhibit GC cell proliferation through regulating miR-144-3p/CBX4 axis.

### CBX4 is upregulated in GC and exerts a promotive effect on GC cell growth in vitro

**Figure 6.**
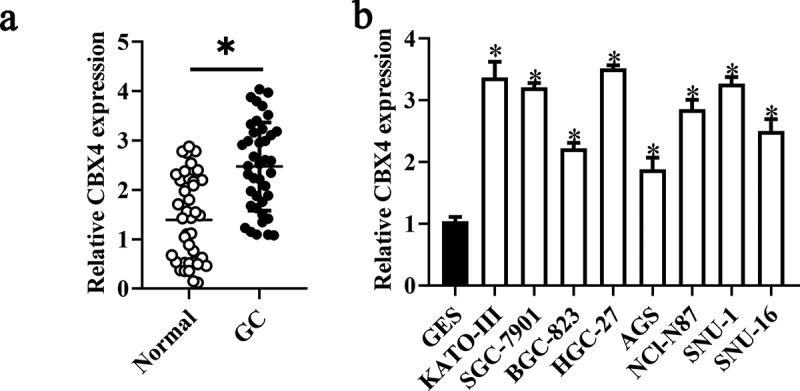
**CBX4 is overexpressed in GC tissue samples and cell lines**. (a) The expression of CBX4 was analyzed in 32 pairs of GC tissue samples and adjacent normal gastric tissues using RT-qPCR. *P < 0.05 vs. the normal gastric tissues. (b) RT-qPCR was performed to test CBX4 expression in eight GC cell lines (KATO-III, SGC-7901, BGC-823, HGC-27, AGS, NCI-N87, SNU-1, and SNU-16) and normal gastric cells (GES cells). *P < 0.05 vs. GES cells

**Figure 7.**
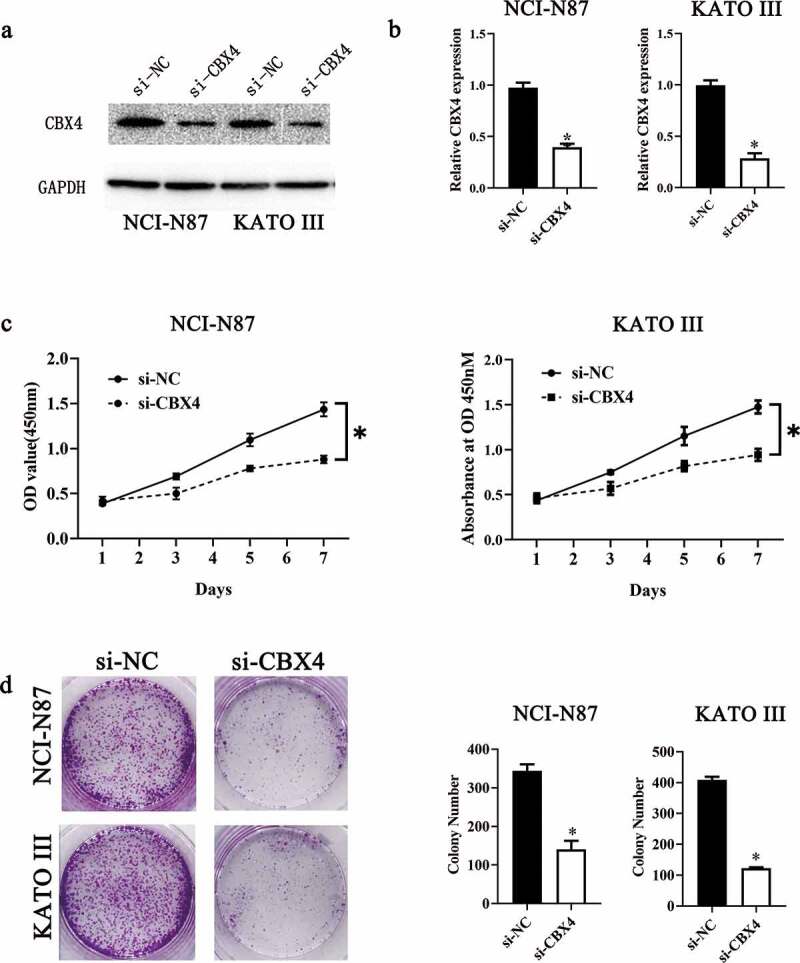
**CBX4 exerts a promotive effect on GC cell growth in vitro**. (a, b) NCI-N87 and KATO III cells were transfected with either si-LINC00265 or si-NC. At 48 h post-transfection, the cells were collected and, then, subjected to western blotting and RT-qPCR analysis for transfection efficiency evaluation. (c) The CCK-8 assay showed a change in proliferation of CBX4-knockdown NCI-N87 and KATO III cells. *P < 0.05 vs. group agomir-NC. (d) NCI-N87 and KATO III cells were treated with either agomiR-144 or agomir-NC. After the transfection, Colony formation assays were carried out. *P < 0.05 vs. group agomir-NC

To further verify LINC00265 knockdown inhibited GC cell proliferation through regulating miR-144-3p/CBX4 axis, we explored the role of CBX4 in GC. For this purpose, we first examined CBX4 expression in 40 pairs of GS tissue samples and adjacent normal tissue samples, as well as GC cell lines and GES cells. CBX4 was found to be overexpressed in GC tissue samples (RT-qPCR; Figure 6a, P < 0.05) and GC cell lines (Figure 6b, P < 0.05). Next, we assessed the effect of CBX4 on GC cell proliferation by silencing CBX4. As shown in Figure 7 A&B, CBX4 was successfully knocked down in NCI-N87 and KATO III cells after transfection of si-CBX4. CCK-8 assays showed that CBX4 knockdown significantly repressed GC cell viability (Figure 7c, P < 0.05). Furthermore, colony formation assays showed silencing CBX4 dramatically reduced the colony formation capacity of GC cells (Figure 7d, P < 0.05). These results showed CBX4 exerted a promotive effect on GC cell growth in vitro, further confirming that LINC00265 knockdown inhibited GC cell proliferation through regulating miR-144-3p/CBX4 axis.

## Discussion

Increasing evidence has suggested that many lncRNAs were dysregulated in GC and played important roles in promoting tumorigenesis and metastasis [33]. Therefore, these lncRNAs may be potential diagnostic biomarkers and therapeutic targets for GC. In this study, we initially tested whether LINC00265 was dysregulated in GC, clarified its effect on GC cell proliferation in vitro and clarified the underlying mechanisms. Our data showed that LINC00265 expression was significantly upregulated in GC tissue samples and cell lines compared to their normal counterparts. Besides, LINC00265 knockdown could inhibit GC cell proliferation in vitro. Further investigation uncovered that LINC00265 functioned as a competing endogenous RNA for miR-144-3p and Chromobox 4 (CBX4) mRNA was as a direct target of miR-144-3p in GC cells. Moreover, inhibition of miR-144-3p could markedly counteract LINC00265 knockdown-meditated suppression on GC cell proliferation.

It has been reported that LINC00265 was upregulated in CRC and played a key role in disease progression. For instance, Ge et al. suggested that increased LINC00265 expression closely correlated with lymph node metastases and advanced pathological stage in CRC, and LINC00265 could promote HT29 cell proliferation and invasion by upregulating the expression of EGFR [37]. Sun et al. also demonstrated that LINC00265 was upregulated in CRC and intimately correlated with poorer patient prognosis. Moreover, they uncovered that LINC00265 could positively regulated CRC cell viability, glucose uptake, pyruvate production and lactate production via the miR-216b-5p/TRIM44 axis [38]. Another study further revealed that LINC00265 was able to regulate ZMIZ2 and USP7-mediated stabilization of β-Catenin, thereby facilitating colorectal tumorigenesis [39]. Interestingly, it was reported that H.pylori can upregulate the expression of EGFR [40] and reduce the expression and activity of intracellular ubiquitinase USP7 in GC cells [41]. Therefore, we boldly hypothesized that H.pylori may induce LINC00265 expression in GC and LINC00265 may contribute to GC progression. Herein, we only focused on the latter due to our financial limitation. As expected, our data showed that LINC00265 was upregulated in GC tissues and cell lines. Additionally, it was observed that LINC00265 knockdown significantly restricted GC cell proliferation in vitro, similar to the previous studies in CRC [37], suggesting that LINC00265 may serve as an oncogene in GC.

We next elucidated the molecular mechanisms underlying the oncogenic action of LINC00265 in GC. Solid evidence has revealed that many lncRNAs can perform biological functions by acting as ceRNAs or molecular sponges to inactivate miRNAs. MiR-144-3p has been reported to be downregulated in cervical cancer [42], ovarian cancer [43], CRC [44] and GC [45], indicating that miR-144-3p may exert tumor suppressive effects in these cancers. For example, miR-144-3p can inhibit GC cell migration by negatively regulating met proto-oncogene (MET) [46] and proliferation [27], and induce cell apoptosis and cell cycle arrest through downregulating COX-2 expression [27]. Strikingly, herein our data showed that LINC00265 served as a ceRNA for miR-144-3p. Moreover, inhibition of miR-144-3p markedly counteracted LINC00265 knockdown-meditated suppression on GC cell proliferation, suggesting that LINC00265 may promote GC cell proliferation by repressing miR-144-3p. Several studies have suggested that CBX4 played dual roles in cancer progression. For example, CBX4 was found to be highly expressed in lung cancer and could promote tumor cell proliferation and metastasis by regulating BMI-1 expression [32,47]. In colorectal cancer, CBX4 can interact with histone deacetylase 3 (HDAC3) to maintain recruited HDAC3 to the Runx2 promoter, which maintains a deacetylated histone H3K27 state to inhibit Runx2 expression, consequently repressing tumor metastasis [48]. With respect to GC, Ying Luo et al reported that CBX4 rs77447679 polymorphism was positively associated with GC, and individuals with CC genotype had lower risk of GC [49,50]. In the present study, we for the first time demonstrated that CBX4 was upregulated in GC tissues and cell lines and CBX4 could promoted GC cell proliferation, indicating it may play a pro-tumor role in GC. Of interest, our further exploration showed that CBX4 was a direct target of miR-144-3p and LINC00265/miR-144-3p axis could regulate CBX4 expression in GC cells.

## Conclusion

In summary, LINC00265 was highly expressed in GC tissues and cell lines. Additionally, knockdown of LINC00265 or CBX4, and miR-144-3p overexpression could inhibit GC cell growth in vitro. Furthermore, LINC00265 could act as a sponge for miR-144-3p to up-regulate CBX4 expression in GC cells. Therefore, LINC00265/miR-144-3p/CBX4 axis may serve as potential promising therapeutic targets for GC[50].

## Supplementary Material

Supplemental Material

## Highlights:

LINC00265 is highly expressed in GC.

LINC00265 acts as a competing endogenous RNA for miR-144-3p in GC cells.

CBX4 mRNA was a target of miR-144-3p in GC cells.

LINC00265 may promote GC cell proliferation via the miR-144-3p/CBX4 axis.
